# Detecting and identifying *Schistosoma* infections in snails and aquatic habitats: A systematic review

**DOI:** 10.1371/journal.pntd.0009175

**Published:** 2021-03-24

**Authors:** Bishoy Kamel, Martina R. Laidemitt, Lijun Lu, Caitlin Babbitt, Ola Liota Weinbaum, Gerald M. Mkoji, Eric S. Loker

**Affiliations:** 1 Center for Evolutionary and Theoretical Immunology, Department of Biology, University of New Mexico, Albuquerque, NM, United States of America; 2 Parasitology Division, Museum of Southwestern Biology, Department of Biology, University of New Mexico, Albuquerque, NM, United States of America; 3 Center for Biotechnology Research and Development, Kenya Medical Research Institute, Nairobi, Kenya; Universidade Federal de Minas Gerais, BRAZIL

## Abstract

**Background:**

We were tasked by the World Health Organization (WHO) to address the following question: What techniques should be used to diagnose *Schistosoma* infections in snails and in the water in potential transmission sites? Our goal was to review and evaluate the available literature and provide recommendations and insights for the development of WHO’s Guidelines Development Group for schistosomiasis control and elimination.

**Methodology:**

We searched several databases using strings of search terms, searched bibliographies of pertinent papers, and contacted investigators who have made contributions to this field. Our search covered from 1970 to Sept 2020. All papers were considered in a PRISMA (Preferred Reporting Items for Systematic Reviews and Meta-Analyses) framework, and retained papers were grouped by technique and subjected to our GRADE (Grading of Recommendations, Assessment, Development and Evaluations) evidence assessment profile determined in consultation with WHO. We also considered issues of sensitivity, specificity, coverage, cost, robustness, support needs, schistosome species discrimination, and relevant detection limits.

**Principal findings:**

Our PRISMA process began with the perusal of 949 articles, of which 158 were retained for data extraction and evaluation. We identified 25 different techniques and for each applied a GRADE assessment considering limitations, inconsistency, imprecision, indirectness, and publication bias. We also provide advantages and disadvantages for each category of techniques.

**Conclusions:**

Our GRADE analysis returned an assessment of moderate quality of evidence for environmental DNA (eDNA), qPCR and LAMP (Loop-mediated isothermal amplification). No single ideal diagnostic approach has yet been developed, but considerable recent progress has been made. We note a growing trend to use eDNA techniques to permit more efficient and replicable sampling. qPCR-based protocols for follow-up detection offer a versatile, mature, sensitive, and specific platform for diagnosis though centralized facilities will be required to favor standardization. Droplet digital PCR (ddPCR) can play a complementary role if inhibitors are a concern, or more sensitivity or quantification is needed. Snail collection, followed by shedding, is encouraged to provide specimens for sequence verifications of snails or schistosomes. LAMP or other isothermal detection techniques offer the prospect of less expensive and more distributed network of analysis but may face standardization and verification challenges related to actual sequences amplified.

Ability to detect schistosome infections in snails or in the water is needed if control and elimination programs hope to succeed. Any diagnostic techniques used need to be regularly verified by the acquisition of DNA sequences to confirm that the detected targets are of the expected species. Further improvements may be necessary to identify the ideal schistosome or snail sequences to target for amplification. More field testing and standardization will be essential for long-term success.

## Author summary

Global efforts are underway to reach the goal of elimination of schistosomiasis as a public health problem by 2030. A crucial step in elimination programs is the verification of elimination, including surveillance of former transmission foci. This systematic review assessed and evaluated a wide range of diagnostic tools for detection of *Schistosoma* parasites in snails and water. Our analysis revealed that along with standard snail shedding methods, molecular methods such as PCR, qPCR and LAMP are becoming the widely adopted standard approaches to detect schistosomes in snails. Recent developments in eDNA methods are further enabling novel surveillance capabilities for snails and schistosomes in water and are likely to become more widely adopted. While there is currently a plethora of techniques to choose from, there is a clear need for further field testing and development of standardized protocol for the most promising among them, including eDNA, ddPCR, qPCR and LAMP methods. Future studies focused on field-worthy detection approaches and their efficacy and sensitivity in the field will be a corner stone in development of control and elimination programs.

## Introduction and background

Schistosomiasis is one of the world’s most prevalent neglected tropical diseases. Although estimates vary considerably, it is generally considered that 800 million people are at risk of infection, with a global prevalence of 229 million cases [1], of which 200 million live in sub-Saharan Africa [2–5]. It has an impact more significant than generally perceived on human health, ranking third among the Neglected Topical Diseases (NTDs) in disability-adjusted life years [6,7]. The *Schistosoma* parasites responsible for causing the disease are found as adult worms in the vascular system of humans and other mammals. The adult worms produce eggs that are passed in the host’s feces in the case of *S*. *mansoni* or *S*. *japonicum*, or the urine in the case of *S*. *haematobium*. The eggs hatch in water and release swimming miracidia that may locate and penetrate an appropriate freshwater or amphibious snail species. Specificity is evident as *S*. *mansoni* successfully infects only *Biomphalaria* snails, *S*. *haematobium* develops in *Bulinus* snails, and *S*. *japonicum* in amphibious snails in the genus *Oncomelania*. Asexual development occurs in sporocysts in the snail host, culminating in the production of fork-tailed cercariae that leave the snail host and swim towards and penetrate unbroken human skin to initiate new infections. Infected snails can produce tens of thousands of cercariae over a period of several months [8].

Schistosomiasis is enabled by poor sanitation, allowing schistosome eggs in feces or urine to pass into snail-containing habitats, and by the widespread use of such habitats for fishing or other occupations, bathing, recreation, washing of clothes, and as a source of drinking water. The long-term persistence of cercariae-producing snail infections in the water renders control more difficult. Even if infected people are successfully treated (usually with praziquantel) to eliminate their adult worms, they may quickly reacquire infections.

The World Health Organization has championed the view that the elimination of schistosomiasis is achievable [9]. In their new roadmap for sustainable development (WHO, 2020), they set a target to validate the elimination of schistosomiasis as a public health problem (defined as <1% proportion of heavy intensity schistosomiasis infections) in all 78 affected countries by 2030. A further goal concerning elimination is to eventually verify and declare the interruption of transmission in a country-by-country manner [10].

Given the importance of development and application of improved diagnostic and surveillance methods for the elimination effort, we have been asked by the WHO to review the suitability of available techniques to answer the following PICO (Problem Intervention Comparison Outcome) question: What techniques should be used to diagnose *Schistosoma* infections in snails and in the water in potential transmission sites? In other words, what are the best approaches for determining the presence and identity of schistosomes in populations of vector snails (frequently known as xenomonitoring), or of snails, schistosome life stages (like miracidia or cercariae) or their DNA in the water of suspected transmission sites? We did not consider literature pertaining to using human fecal bacteria as surrogates for assessing the likelihood of schistosome contamination of surface waters [11].

Such methods provide a needed alternative view to diagnosis or surveillance of infections in the human host. Snail or water-oriented methods might uniquely detect transmission if, for instance, eggs from non-human reservoir hosts were responsible for infecting snails. Monitoring events in snails or in the water also helps determine if particular schistosome species have been introduced into new areas or re-introduced into former endemic areas, define transmission hot spots spatially and temporally, and gauge the success of intervention methods targeted at the human population [12,13]. The successful effort to control *Schistosoma japonicum* in China has been aided by monitoring infections in snails and in the water of suspected transmission sites [14]. Considerations important to our evaluation include the points listed in **Table 1**.

**Table 1:** The ideal diagnostic test to answer the stated PICO question should include the following characteristics.

• Be specific in the sense of correctly detecting the target schistosome or snail species in the specific country/geographic area under consideration
• Be sensitive such that false negatives are avoided even in areas where low levels of infection are expected (particularly important for elimination considerations)
• Take into consideration that infections in snails may be pre-patent at the time of sampling, meaning they have not yet developed sufficiently to produce cercariae
• Be robust to using samples from different kinds of habitats or snail species
• Take into consideration geographic (and possibly sequence) variability of the target species
• Be scalable such that the degree of spatial and temporal coverage is sufficient (and possibly allow for pooling of samples if needed)
• Be able to be checked by a method like sequencing to validate identifications
• Be periodically subjected to independent quality control
• If possible, return quantitative results as opposed to simple present/absent results
• Allow for documentation of the sampling effort in the form of specimens that can be contributed to curated museum collections for confirmation of identities of snails or schistosomes examined and for future study
• Be fast, not labor intensive nor demanding of high levels of technical skills
• Employ standardized sets of reagents and instrumentation to the extent possible
• Be robust to variability due to sampling and processing by different lab groups
• Not require complex or expensive protocols or instrumentation
• Not impose complex storage demands

**Fig 1:**
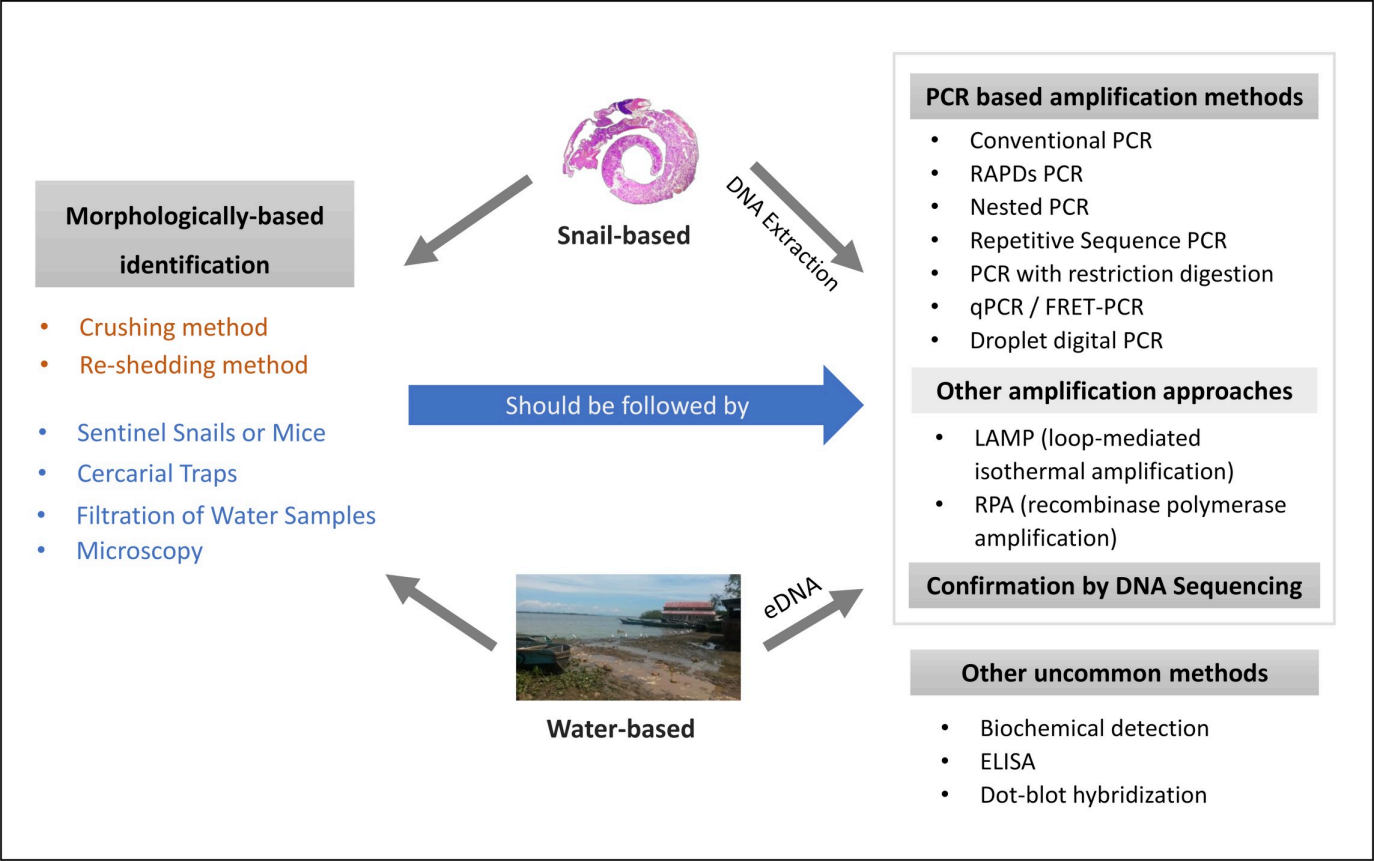
Shown are methods for detecting schistosome infections in snails or for detecting schistosome or snail signals in the water. Identification protocols rely on traditional morphological means or, increasingly, sequence-based identifications. With respect to finding infections in snails, the traditional technique of isolating and shedding snails for cercariae is widely practiced, but alternative methods, mostly relying on amplification of schistosome DNA sequences by a variety of means, is also widely practiced. Additional follow up techniques may be required to provide sufficient sequence information for identification. A variety of techniques has been devised to detect schistosomes in the water column ranging from using sentinel mice to collect cercariae from the water to collection of environmental DNA samples containing DNA from intact or disintegrated bodies of schistosomes or their snail hosts. Again, follow-up sequencing may be required to confirm species identifications.

A detailed list of the general diagnostic procedures we considered is provided in the results section. For those who may not be familiar with the general approaches taken over the years to detect and identify schistosome infections in snails or in the water column, we provide an overview in **Fig 1** of the available diagnostic approaches that have been undertaken relative to our PICO question.

## Methods

### PRISMA process

#### Inclusion guidelines

Following the PRISMA guidelines [15], we examined published papers and other materials primarily to do with human-infecting *Schistosoma* species wherever they might occur, from 1970 to Sept 2020, from WHO’s six official languages. Where relevant papers existed, as with the study of cercarial dermatitis or fascioliasis in snails, we have examined those papers as well.

#### Exclusion guidelines

We excluded from this analysis papers published before 1970. We also excluded papers that were essentially reviews that did not include new data about diagnostic techniques. We also excluded papers dealing with the diagnosis of schistosome infections in humans for which an extension of the technology to other contexts did not seem likely. We also excluded papers for which the full text was not available or insufficient data were present to evaluate.

#### Information sources identified

We searched PubMed, Web of Science, Google Scholar, China Academic Journals Full-text Database, Mendeley, and ResearchGate using the combination of search terms listed below. Particularly ResearchGate held papers that might not have been indexed by the major search engines. We also searched the reference lists provided in the articles or reports we found that were most germane to our PICO question. We contacted 11 experts and previous prominent contributors to this literature by email and asked for any unpublished or recent reports not yet available on search engines. We viewed relevant YouTube videos bearing on our PICO question. By persistently pursuing the “literature cited” sections of the papers we examined and adding papers that had not yet come to light, we eventually did not find any new papers that had not already turned up multiple times in our various search processes.

#### Search strategy

Each query was executed independently. The search strings were designed using logical operators to encapsulate terms related to schistosomiasis and the various monitoring techniques. In addition, search terms included related parasite species to capture papers that hold methods suitable for schistosomiasis detection in snails or the environment.

Schistosoma AND DETECTION AND (PCR OR SENTINEL OR LAMP OR XENOMONITORING OR SHEDDING OR RTPCR OR DDPCR OR qPCR OR SURVEILLANCE) AND SNAIL

Bilharzia AND DETECTION AND (PCR OR SENTINEL OR LAMP OR XENOMONITORING OR SHEDDING OR RTPCR OR DDPCR OR qPCR OR SURVEILLANCE) AND SNAIL

Schistosomiasis AND DETECTION AND (PCR OR SENTINEL OR LAMP OR

XENOMONITORING OR SHEDDING OR RTPCR OR DDPCR OR qPCR OR SURVEILLANCE) AND SNAIL

Trichobilharzia AND DETECTION AND (PCR OR SENTINEL OR LAMP OR XENOMONITORING OR SHEDDING OR RTPCR OR DDPCR OR qPCR)

Schistosoma AND Filtration AND (PCR OR SENTINEL OR LAMP OR XENOMONITORING OR SHEDDING OR RTPCR OR DDPCR OR qPCR OR SURVEILLANCE)

Trichobilharzia AND Filtration AND (PCR OR SENTINEL OR LAMP OR XENOMONITORING OR SHEDDING OR RTPCR OR DDPCR OR qPCR OR SURVEILLANCE)

Trap AND Detection AND Schistosome

#### Inclusiveness

We interrogated databases independent of the language content of the titles and abstracts. The team writing this report has members proficient in Chinese, French, Spanish, Arabic, and English. We examined all possible literature available on our PICO question in these different languages if it existed.

#### Reference database construction and management

Search results from each query were exported in RIS/BIB/MEDLINE format, merged, and imported into the open source reference manger JabRef [16]. The database was then de-duplicated, using the built-in de-duplication function in JabRef. Some missed duplicates were then manually removed. Records that included authors from the team preparing this report were noted and are highlighted with an asterisk.

#### Additional sources

References obtained by contacting authors directly were added to the database.

#### Inclusion/exclusion criteria

We examined the combined non-redundant database of all search outcomes. Despite the specificity of our search strategy, many articles dealt with the detection of schistosome infections in human subjects and were excluded unless some connection to snail or water-related research was evident. Articles dealing with social or economic aspects of schistosomiasis were deemed irrelevant to our question and excluded. Papers describing differentiation techniques of various schistosome species or, in a few cases, snail species were included in the analysis. Any articles presenting a method for the detection of schistosomes in snails or water bodies were included.

#### Data extraction

Using the predefined template shown in **S1 Table,** we set out to extract information from all articles included in the analysis by the criteria described above. The full text of each article was downloaded, and additional articles in press or in review were obtained from authors. Interlibrary loan was used to retrieve articles unavailable on the web. The results of the data extraction were agglomerated in a shared spreadsheet accessible to the whole team, and the comments provided reflect our collective input.

#### Full-text exclusion/inclusion criteria

During the process of data extraction from the full-text articles, some additional articles were marked for exclusion from our final PRISMA tally if it was determined they only dealt with human subjects, no new data were included, or we were unable to obtain the full text.

#### Determination of certainty of evidence following GRADE criteria

We were asked to determine the certainty of evidence or confidence in effect estimates using the GRADE approach used in clinical medicine and public health policy [17]. The studies we evaluated had different designs, employed a range of techniques, and targeted different schistosome species or sequences for amplification. Because these studies did not focus on a single effect, a formal meta-analysis was not undertaken. Given the lack of data in the randomized control trial format for the studies we reviewed, we consulted with WHO before initiating a modified GRADE evidence assessment using the following criteria:

***Limitations*** *(risk of bias)*. These were first considered for each study, then evaluated for all studies grouped together using a specific technique. This involves assessing the use of an unbiased approach for measurement of outcomes, using adequate controls, and asking if there are apparent confounders. We included criteria relevant to our PICO question including an adequate number of samples examined to make a judgment; the adequate number of different conditions tested (water, reagents); quality of methods and equipment used to obtain results (molecular procedures, microscope, gel rigs, etc.); the amount of sample required; the amount of time to process samples; and use of proper controls. This also included questions like if gel bands were sufficiently sharp, bright and measurable, output was quantifiable and comparable between groups and correct identification of schistosomes down to the level desired (species level, hybrid level) was achieved.

***Inconsistency***. Were results variable across studies (do points of view vary widely between studies), or were there non-overlapping confidence intervals among studies? We also asked if there is day-to-day variability, variability between labs, or if the technique is “finicky” (overly dependent on specific reagents or successful only in a handful of reports).

***Imprecision***. This considers if data are sufficiently precise to adequately analyze or make a correct conclusion from it, and associated power of statistical analysis. Imprecision refers to the size of the evidence (adequate number of samples, sufficiently narrow confidence intervals).

***Indirectness*.** For our consideration, if the method did not apply to real-world practice, it would be labeled as indirect. For example, if a particular method requires overly complex equipment or hard-to-get reagents.

***Publication bias***. do published studies differ substantially from unpublished studies (gray literature) in showing an effect? For example, are there indications that negative results are being suppressed?

Furthermore, we upgraded the quality of GRADE evidence for observational study results if 1) dose-response gradient data were provided; 2) if the study persistently handicapped itself by the inclusion of the most severe cases needed to document an effect; and 3) if the magnitude, precision or consistency of the effect reported was deemed strong.

In addition to these five GRADE criteria, for our specific PICO question, we also considered some additional points deemed to be relevant, as follows:

***Sensitivity of detection***. For our PICO question, the issue of “sensitivity” needs to be considered in a way different from how it might normally be conceived. For example, it is relevant to know if the method can detect early/immature infections in the snail or light infections. Failure to detect them would be considered false negatives. Molecular methods usually define sensitivity as the minimum amount of DNA needed for a positive result. Other methods that rely on recovering the actual parasites depend on the concentration of parasites per snail or specified water volume. Some methods combine both the collection of parasites and molecular detection limits, such that sensitivity is related to both abilities to concentrate parasite material and the minimum DNA amount possible for molecular detection. We have identified the following categories of “detection limits” as the best way to consider “sensitivity” relative to our task.

***For snail-based techniques***: *High sensitivity*—ability to detect snail with one parasite at 1-day post-infection, or the ability to detect prepatent infections two weeks or younger in duration; for pooled samples, one positive snail among a pool of 100 snails. Marginal sensitivity–the ability to detect prepatent infections >2 weeks old or patent infections. Inadequate sensitivity—inability to reliably detect snails with patent (shedding) infections.

***For water samples***. High sensitivity—ability to detect the equivalent of one cercaria or less in a 20-liter water sample. Marginal sensitivity—ability to detect the equivalent of 5 cercariae per 20-liter water sample. Inadequate sensitivity—inability to detect > 5 cercariae (or equivalent DNA amounts) in a 20-liter sample of water. This information was not reported in all the studies we evaluated. Therefore, we had to base our judgments about a particular technique on those studies that did specify such information or used other parameters as a proxy to judge the sensitivity, such as the amount of DNA, or amount of starting material needed for a positive signal.

***Species differentiation***. We considered if the technique is specific enough: to tell species within a species group apart (for example, *S*. *mansoni* from *S*. *rodhaini*, or *S*. *haematobium* from *S*. *bovis*); possibly to tell hybrids from non-hybrids, and to not provide false positives for snails with infections with non-schistosome trematodes. Each method can be judged as: *Monospecific*—verified to detect the target species in question only (e.g. only *S*. *haematobium*); *Narrowly heterospecific*—detects the target species but cannot differentiate close relative (e.g. *haematobium*, also detects other members of *S*. *haematobium* species group like *S*. *bovis* or *S*. *mattheei*); *Heterospecific*—records *Schistosoma* of multiple species groups (e.g. *S*. *mansoni* and *S*. *haematobium*); *Nonspecific*—not clear from the literature if the method can reliably detect *Schistosoma* species at the exclusion of other digeneans species or other organisms with ample confidence.

***Coverage***. How much labor is needed to permit the collection, preparation, and analysis of samples required for a specified technical approach? Can adequate coverage of habitats be achieved to facilitate sound judgment?

***Cost***. Cost involved for specialized training, reagents, computer analysis or processing time. In most cases, the cost was provided by the authors in the paper. When that was not discussed, we compared the prices of reagents required to current known prices. In complex methods, we considered the cost of equipment and reagents needed to execute the particular method.

***Support needs***. Requirements for extensive lab space, rearing facilities, electrical service freezers, distilled water, specialized instrumentation, and service contracts were considered.

***Pros and cons*.** As a more intuitive overview of our deliberations, we include a table that highlights the perceived pros and cons of each technique we considered.

## Results

### PRISMA flow diagram

**Fig 2:**
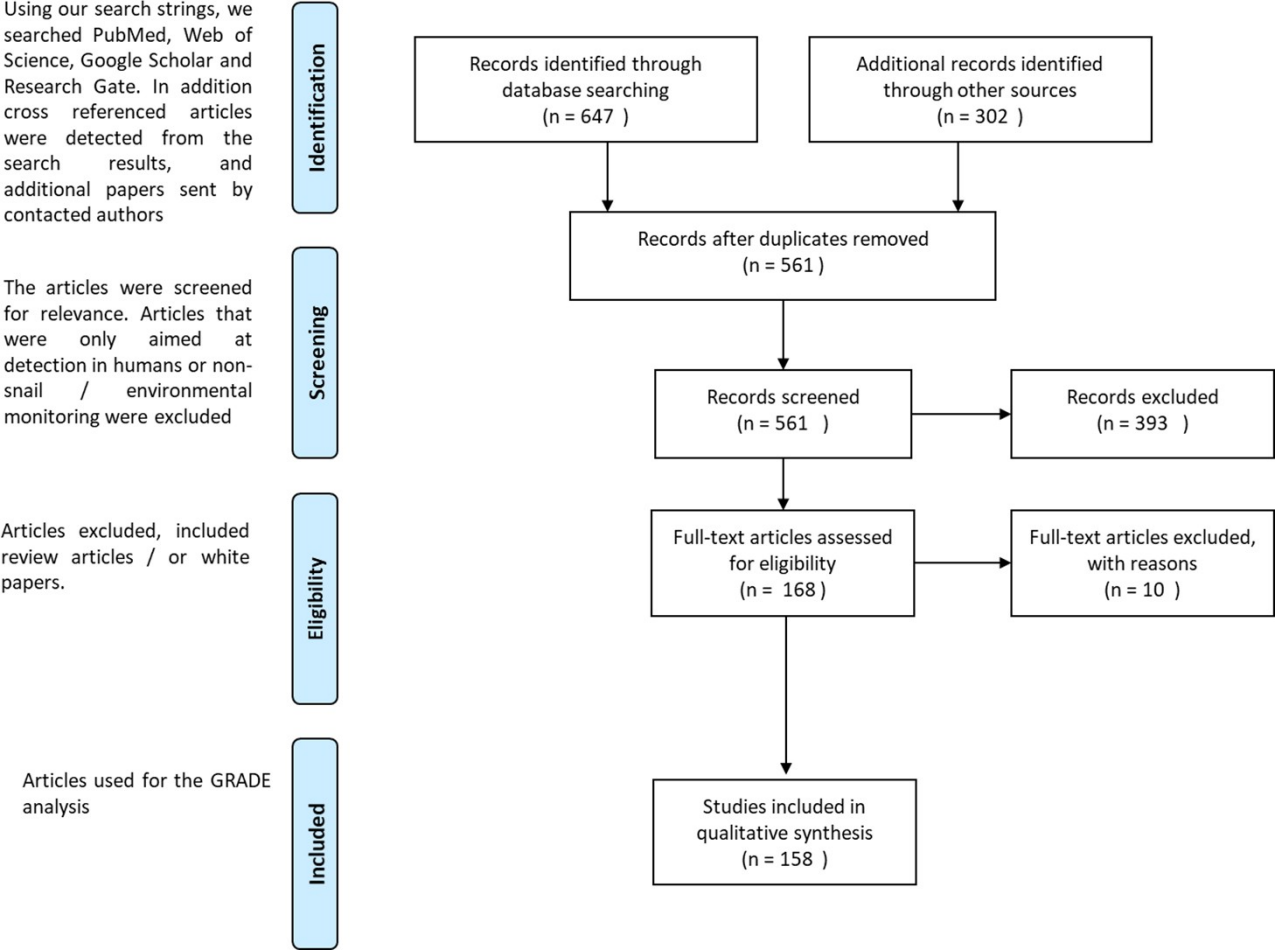
Overview of the PRISMA process.

The Prisma diagram (**Fig 2**) provides an overview of how we arrived at the final 158 articles included in our systematic review process.

### Papers reviewed, and our extraction summaries

**S1 Table** provides the 158 articles along with our extraction summaries that were considered in our GRADE assessments. Three of the articles we examined were authored by one or more members of our team, so an asterisk has singled out these articles in the table.

### Determination of certainty of evidence following GRADE criteria

We grouped the techniques covered in the 158 papers into 25 categories (**Table 2**). Some papers discussed more than one technique. As noted in **Fig 1**, the techniques cover a wide range of approaches. Some are directed towards detecting schistosome infections in snails, and some involve the detection of schistosomes (or snails) in water samples. In some cases, like shedding of snails, identifications of schistosomes can be undertaken using morphological criteria or by submitting them to molecular techniques. For some methods like eDNA, the process typically involves collecting a sample followed by extraction and the submission of the sample to some type of amplification protocol. The data supporting the scores provided in **Table 2** are provided in **S1 Table.** The five criteria contributing to the overall certainty in the grade score provided were limitations, inconsistencies, imprecision, indirectness, and publication bias. As we did not detect the latter, it does not appear in the table. The remaining five columns (coverage, cost, support needs, species differentiation, and relevant detection limits) reflect our view of their importance, but they were not included in the GRADE scores. Note that by the GRADE criteria provided, many techniques were assessed as having very low or low overall certainty: in several cases, they reflect techniques reliant on older technology. Three techniques were scored as providing moderate certainty: LAMP, eDNA based techniques, and qPCR. One of the significant considerations diminishing confidence for several of the techniques was the lack of widescale testing and standardization considerations.

**Table 2:** Grade Summary Table. We used the numbers 0, -1, -2 to score every method, with 0 being adequate, -1, serious concern, -2 very serious concern. It is worth noting that publication bias is not part of this table, as we did not have any reason to suspect it has occurred with any of the methods. We defined species differentiation ability of each method as **N.S** = Nonspecific, **H.S** = Heterospecific, **N.H.S** = Narrowly Heterospecific, **M.S** = Monospecific. And relevant detection limits as **Hig.S,** High sensitivity**, Mar.S** = Marginal sensitivity**, Ina.S =** Inadequate sensitivity.

Method	Number of studies	Limitations	Inconsistency	Imprecision	Indirectness	Overall certainty	Coverage	Cost	Support Needs	Species differentiation	Relevant detection limits
Direct Shedding	20	-2	0	0	0	Low	0	0	0	H.S	Mar.S
Snail crushing	17	-2	0	0	0	Low	-1	0	0	H.S	Mar.S
ELISA/immunodetection	7	-1	-1	0	-2	Very Low	-1	-2	-2	H.S	Mar.S
Biochemical analysis	2	-1	-1	-1	-2	Very Low	-1	-2	-2	N.S	Mar.S
DNA hybridization /DOT BLOT	5	-2	-2	-1	-1	Very Low	-1	-2	-2	H.S	Ina.S
**PCR**											
Conventional PCR	39	0	-1	-1	0	Low	-1	-1	-1	M.S—H.S	Hig.S
PCR with Restriction digestion	2	-1	-2	0	0	Very Low	-1	-1	-1	M.S—H.S	Hig.S
RAPD PCR	1	-2	-2	-2	0	Very Low	-1	-1	-1	H.S—N.S	Mar.S
Repeat Sequence PCR	7	-1	-1	-1	0	Very Low	-1	-1	-1	H.S—M.S	Hig.S
Nested PCR	7	0	-2	0	-1	Very Low	-1	-1	-1	M.S	Hig.S
Multiplex PCR	8	0	-1	-1	0	Low	0	-1	-1	M.S	Hig.S—Mar.S
qPCR	17	0	0	0	-1	Moderate	0	-1	-1	M.S	Hig.S
FRET-PCR	5	0	0	0	-1	Moderate	-1	-1	-1	M.S	Hig.S
**ddPCR**	2	-1	-1	0	0	Low	-1	-2	-2	M.S—H.S	Hig.S
Isothermal Amplification Techniques											
LAMP	23	0	-1	0	0	Moderate	0	0	0	N.H.S	Hig.S
Microfluidics LAMP	1	-1	-1	0	-2	Very Low	-1	-2	0	N.H.S	Hig.S
Recombinase polymerase amplification	3	-2	-1	0	0	Very Low	-1	-1	0	N.H.S	Hig.S
**Water-based detection**											
Filtering then direct exam of filter	6	-1	-2	-2	0	Very Low	-1	-1	0	N.S	Mar.S
Sentinel rodents	15	-1	-1	-1	-1	Very Low	-1	-2	-2	H.S	Hig.S
Sentinel snails	5	-1	-1	-1	-1	Very Low	-1	-1	-1	H.S	Hig.S
eDNA	10	0	-1	0	0	Moderate	0	-1	-1	N.H.S—M.S	Hig.S
Cercariae traps	4	-2	-2	-1	0	Very Low	0	0	0	N.S	Mar.S
Robotics	1	-1	-1	-1	-1	Very Low	0	-2	-2	N.S	Ina.S
Others*											
Oligochromatic dipstick	2	-1	-1	0	-1	Very Low	-1	-2	0	M.S—H.S	Hig.S
Filtration then molecular characterization	1	-1	-1	-1	0	Very Low	-1	-2	0	M.S—N.S	Mar.S—Hig.S

*These two techniques are used in conjunction with other methods on the list, but both warranted special mention because each is technically distinct from the methods with which they are usually coupled.

In **S2 Table,** following GRADE criteria, we present determinations of sensitivity and specificity using the definitions often applied to diagnostic techniques in the medical literature (see table legend for definitions). Insofar as most of the papers we reviewed simply did not provide this information, its relevance to our discussion is lessened. Lastly, in **Table 3**, with an eye on practical guidance, we provide an overview of the pros and cons of each of the techniques we classified.

**Table 3:** A summary of perceived advantages and disadvantages of the techniques evaluated, with comments on possible improvements and potential applications.

Techniques	Pros	Cons	Improvement needed	Potential applications
**Direct Shedding**	- A low tech and widely familiar and practiced technique with the advantage of providing a powerful indicator of ongoing transmission and infection - Cost within financial means of most labs - There is a broad basis of use in many labs for many years - Provides actual specimens for further study and archiving - Provides important training component for field workers	- Can miss prepatent infections unless special precautions are taken like repeat shedding that in turn require snail-holding facilities - Requires specialized training in snail and especially cercariae identification - It is unlikely by itself to provide a complete picture of transmission but also hard to envision a surveillance program that does not involve some direct examination of vector snails - Identifications will require molecular confirmations - Poses a risk of exposure of investigators to numerous cercariae, so carries biosafety requirements	- Basic levels of malacological and associated parasitological training need to be encouraged - Specimens collected are invaluable but must be reliably and safely handled and labeled and subjected to further curation and identifications, both for putative schistosome vector snails and the schistosome cercariae found	- Maybe a necessary accompaniment to any technique relying only on the detection of sequences from the environment - By providing actual specimens of organisms that can be curated, it offers use in future applications of techniques we cannot presently foresee
**Snail Crushing**	- Is a follow up to snail collecting - Can be used instead of shedding to screen snails - Works better with small snails like *Oncomelania* - Low tech and within the means of many field labs - It has the advantage of finding both patently infected snails and at least some prepatent infections	- With bigger snails with early prepatent infections, it is likely that infections would be missed - Early infections of schistosomes may be hard to differentiate from those of other species - In many geographic contexts, discrimination among schistosome species would require further application of molecular techniques - It destroys the physical integrity of the snails collected	- There may be ways to increase the value of this approach by pooling of snails or fluids from them (would work better for smaller snail species) and then screening a pooled extraction using various molecular approaches - Perhaps such pooling could be done with just those snails found to have intramolluscan larvae that could not be identified with certitude as being schistosomes	- Could play a particularly useful role to complement eDNA approaches that do not provide actual specimens
**ELISA/ Immunodetection**	- Lends itself to a high-throughput format, which is established for many ELISA assays - Can be very sensitive and specific in particular contexts	- The ability to distinguish among closely related schistosome species has not been rigorously tested - The needs for ELISA reader and specialized monoclonal antibodies would likely preclude use in most field labs - Not a lot of current technique development	- Many improvements are theoretically possible, but it seems unlikely ELISA-based techniques will soon displace sequence-based detection methods with the ability of the latter to confirm species identities	- It is possible particular antigens characteristic of schistosomes will prove to be abundant and stable in snails or water samples to warrant further ELISA-based detection
**Biochemical Analyses**	- Some approaches could be adapted to high-throughput	- Lacks specificity needed to be sure of parasite species identification - Cumbersome and expensive for most field labs - General lack of any widespread field testing or current development	- Unlikely forthcoming given other options already available	- Not presently foreseen given other alternatives
**DNA hybridization/ DOT BLOT**	- This was the original application of DNA-based technology in the context of the PICO question	- Is hybridization-based - Making probes is cumbersome - Blotting process and readout of spots not nearly as precise as gel bands of PCR products - Issues of quantification, sensitivity, and specificity are harder to gauge accurately	- The appearance of this technique heralded new emphasis on the use of DNA in diagnosis and stimulated innumerable improvements in sequence-based nucleic acid-based detection, but hybridization per se as detection system has not been emphasized	- Extensive developments in DNA-based diagnosis in the intervening years make it unlikely this hybridization-based technique will be extensively used in the future
**Conventional PCR**	- Often sensitive and specific - Cost and time per sample is coming down, lower than qPCR or ddPCR - Can be coupled with simpler and less expensive extractions - Is widely used, and equipment becoming more universally available - More convergence on standardized reagents, protocols, and targets is possible	- Requires gel to visualize products - Depends on the availability of thermocycler and specialized reagents - Subject to contamination without precautions - Specificity needs to be checked by sequencing - Standardization is a concern	- Periodic verification of the nature of amplicons by sequencing is desirable and necessary to support vital conclusions - Less expensive reliable thermocyclers are needed and becoming more available - Standardization of platform across laboratories is a desirable goal	- Has been and will likely continue to be an important starting point for many innovative diagnostic techniques going forward
**PCR with Restriction Digestion**	- Can be sensitive and specific - Might be able to resolve closely related schistosome or snail species	- Amplicons generated may vary with geographic isolates and have different restriction patterns - The same considerations that apply to conventional PCR apply - Cumbersome in requiring restriction digest follow-up and gel run and need to accurately size bands - Identity of amplified bands is often unknown	- It seems unlikely this approach will receive additional development to enable standardization and simplification	- In light of the development of newer technical approaches, it seems unlikely to play a major role but might help to resolve closely related species in some cases
**RAPD PCR**	- Provides a distinctive profile of randomly amplified bands that might enable more specific detection of a certain species - Can be sensitive	- Less likely to produce consistent band patterns, especially across broad geographical regions - Identity of amplified bands is unknown unless followed by explicit sequencing - Largely replaced by more repeatable primer specific driven amplification	- It seems unlikely this approach will receive additional development to enable standardization and simplification	- In light of more specific primer and sequence driven approaches, it seems unlikely RAPDs RCR will play a major role in future diagnostic efforts
**Repeat Sequence PCR**	- Sensitivity is high, at least in some cases as with *S*. *haematobium* group - Highly repetitive sequences have an inherent appeal because of their abundant representation in the schistosome genome - They have been used by more studies than any other PCR targets	- Depends on the availability of thermocycler and specialized reagents - The same considerations that apply to conventional PCR	- Specificity maybe not sufficient for distinguishing among closely related species, but workarounds are being devised - May also be a need to check for distantly related but nonetheless cross-reacting species	- This approach has been used in real-life surveillance context, but some additional specificity testing in demanding field contexts is warranted - Compatibility with eDNA based sampling needs to be checked
**Nested PCR**	- Very sensitive because of the nested approach - Specific because of a nested approach	- Depends on the availability of thermocycler and specialized reagents - Cost and time per sample higher than with conventional PCR because of the need for two successive runs - Contamination a concern - Still a need for periodic verification by sequencing	- It seems unlikely nested protocols will be needed to achieve desired sensitivity or specificity, so further development is not expected, especially in view of probable further development of ddPCR	- It might have some specific application in situations where originating signals are weak, and more sensitivity is needed
**Multiplex PCR**	- Multiplexing is a potentially useful adjunct for many of the DNA amplification-based protocols listed here - Can be sensitive and specific - Can reduce labor and reagent expenses	- Requires thermocycler and specialized reagents and mutually compatible primers - Still dependent on periodic verification of amplicon identity by sequencing - May be difficult to get it working consistently, especially across different labs - The usual concerns about contamination apply	- Efforts to standardize extraction, reagents, primers, and protocols may improve the consistency of multiplexing results across different labs	- Has potential to simultaneously resolve the identity of multiple species (possibly multiple schistosome species and/or snail vector species as well) - Could play a key role if coupled with versatile sampling and amplification protocols
**qPCR**	- Relatively quantitative - Can be more sensitive and specific than conventional PCR - Mature technique supported by extensive commercial development - Amenable to high-throughput and multiplexing with less time and lower costs per sample - No follow-up gel run required - When coupled with labeled probes can provide specificity, requiring less follow-up sequence verification	- Depends on the availability of RT-PCR machine requiring calibration and specialized reagents - Can be expensive if runs are limited - Some follow-up sequence verification desirable - Contamination can be an issue but less so than with conventional PCR - May be necessary to overcome inhibitors - Standard curves and triplicate runs need to be made	- More portable and less expensive devices becoming available - More testing with a great variety of samples is needed - More standardization of methods and reagents is desirable	- Has become a method of choice coupled with eDNA collection protocols - Once set-up and standardization achieved, it can be high-throughput - May be necessary to set up designated centers to run many samples - ddPCR may assist with inhibitor or sensitivity problems
**FRET-PCR**	- See comments on qPCR as well - Relatively quantitative - Can be very sensitive and specific - Mature technique supported by extensive commercial development - Amenable to high-throughput with lower costs per sample - Provides species-specific detection - Less verification of the identity of amplicons by sequencing needed than with qPCR	- Depends on the availability of RT-PCR machine requiring calibration and specialized reagents - Can be expensive if runs are limited - Contamination an issue but less than conventional PCR - May be necessary to overcome inhibitors - Standard curves need to be made	- More portable and less expensive devices becoming available - More testing with a great variety of samples needed - Standardization of methods and reagents is desirable	- Has become a method of choice coupled with eDNA collection protocols —ddPCR may assist with inhibitor or sensitivity problems - Has been shown to work to detect schistosomes in snails, human stool samples, and water
**ddPCR**	- Offers absolute quantification - Resilient to the presence of inhibitors which are diluted - Less prone to PCR contamination - Can be more sensitive than other amplification protocols - Primers allow for the potential of specificity	- Requirements of specialized and expensive ddPCR machine - Currently, techniques including emulsification not standardized nor widely tested for field conditions - Lack of testing for snail or aquatic samples thus far - Backup checking for specificity desirable	- Efforts to make it less expensive with more standardized protocols will be welcome - More experience in detecting schistosomes in snails or water samples from a variety of sources needed	- Offers promise for accurate quantification - May serve as needed confirmatory back-up technique for other techniques like qPCR or LAMP
**LAMP**	- Simple, low-tech isothermal amplification conditions - Simple, fast readout not requiring gel running - Sensitivity often as good as or better than PCR - Efforts developed to provide standardized and easily stored reagents and SYBR-based detection methods not requiring specialized instrumentation - Extraction can be inexpensive - Can be coupled to long-term storage of extracted samples on filter paper	- Specificity, consistency, and optimization needs more attention, especially in comparison with comparable PCR-based approaches - Sequence verification periodically required - Amplified products not as simple to sequence - Not quantitative - Can be subject to contamination - Visualized products convey relatively little information	- Standardization of reagents and conditions and optimization to make the technique more robust and comparable from lab to lab - Improvements to provide better readout of results to enable quantification of desirable targets - Improvements to facilitate follow-up sequence analysis would increase confidence in the identity of amplicons	- Offers the prospect of a distributed low-cost network of analysis of samples as opposed to a more centralized facility dependent on more elaborate instrumentation
**Microfluidics LAMP**	- For a certain volume of water, it might be more cost-effective - Its use could facilitate standardization across labs and enable some poorly equipped labs to provide results equivalent to those of other labs	- There is a concern about the expense of achieving sufficient coverage - For an individual snail, it would be prohibitively expensive	- Technique has considerable potential with more development but not yet adequately tested - Costs likely to be prohibitive for wide-scale use for some time - Pooling of samples may help offset expenses	- This specific technique has not been developed to detect *Schistosoma* in snails or in the water, so it might have a role to play, particularly in distributed analysis networks
**Recombinase polymerase amplification**	- Simple isothermal amplification conditions and associated equipment - Enables rapid readout of results at the point of need, including on a chip	- As yet relatively untested as compared to other amplification methods - Requires proprietary reagents - Sequence verification of products required periodically - Transfer contamination possible	- Further development is encouraged to assess applicability to snail or water detection - Vulnerability to inhibitors or contamination and standardization across locations remain to be determined	- Only used thus far in definitive host stages but has potential for detection in snails and could play a role in distributed networks of analysis
**Filtering then direct exam of filters**	- Cercariae stained and observed directly on filters - Actual costs of filters and apparatus can be very low with low-tech gravity filter set-ups - Can be integrated with other data like time of collection to reveal transmission dynamics	- Samples may be inconsistent in quality because of variation in water turbidity from location to location - Labor intensive to collect water samples, filter them, and then microscopically screen filters - Coverage limited by labor - Cannot provide definitive identification so would require follow-up molecular identifications in many areas, as in most of sub-Saharan Africa	- The essential improvement has perhaps already been made (extracting DNA from the filters rather than looking for actual parasite specimens on them) - Less labor-intensive or more efficient alternatives to filtration to collect samples would be desirable	- Experience gained in filtering water and then collecting filters may prove to be useful to developers of eDNA based techniques (it may be easier, for example to use filter-free collection techniques)
**Sentinel rodents**	- It can be very sensitive in detecting the presence of transmission in specific locations - Provides vivid indicator of lingering transmission - Provides actual specimens of worms amenable to many different kinds of studies, and for archiving - It is relatively low tech to maintain mouse colonies	- Ethical concerns about the use of numbers of mice often required - Requires animal care and use committee approvals - Hard to get widespread coverage because of escalating mouse numbers - Takes weeks to see results, and mice may die in the interim - Specific identification of recovered worms may require additional molecular follow-up in some locations	- Its use might be better justified if other techniques were to highlight specific locations where more direct evidence of danger of acquiring infection was required - It might highlight the need to trap and examine wild rodent reservoirs of schistosomes associated with particular locations	- May be useful in particular circumstances where any threat of existing transmission urgently needed to be identified - It provides direct evidence that a schistosome infective to rodents is present - Would facilitate the establishment of a life cycle of schistosome in the laboratory if this was needed for some reason - Useful for *S*. *japonicum* with cercariae that are sticky and hard to process as on filters
**Sentinel snails**	- It can be a sensitive and unique way to detect and measure transmission mediated by humans or other definitive hosts to snail vectors	- It requires a large colony of lab-reared snails to provide uninfected sentinel snails - Care required to avoid accidental snail releases - Difficulties of specific identification of any schistosome-positive snails are similar to those encountered with shedding methods - Have to wait for the infections to develop until detectable - Snails may be prone to die in the field or when brought back to the lab - Coverage usually limited	- Improvements in mass snail rearing, maintenance, and screening might make this technique feasible, especially in small or limited transmission foci	- Could be scaled up to provide a distinctive overview of transmission but would require resources to support extensive snail colony - Some potential for this technique to trace the dynamics of transmission and distributions of infections among snails, particularly if the suspected transmission site is small
**eDNA**	- It allows rapid collection of large numbers of samples, so facilitates habitat coverage - Samples likely integrate parasite signals over time and space, thus broadening coverage range - Lends itself well to qPCR or ddPCR - It has become a popular approach, so will receive considerable attention for the development - Can yield surprising results and detect the presence of organisms missed by classic survey techniques	- By itself does not produce a result (eDNA sample must be extracted and used in conjunction with a molecular detection or identification method) - More testing in a variety of different habitats and situations is needed, including to assess rates of degradation and movement of eDNA in relevant natural habitats - More standardization, including the best associated molecular technique to use (LAMP, qPCR, ddPCR), is needed - It is not known if positive samples are due to the presence of living miracidia or cercariae or relict DNA derived from them	– Further study is needed to determine the stability of samples on filters post-collection - Susceptibility of eDNA to contamination upon subsequent amplification attempts requires more investigation - Simpler ways of sample collection other than filtering water deserve scrutiny - Development of high-throughput processing of samples would take advantage of the greater potential sampling coverage of this technique - May be possible to develop eRNA techniques to discern the presence of living parasites	- Use of eDNA has already shown potential for pragmatic detection of schistosomes or snails in water samples - Offers hope for more precise location of hot spots and broader habitat coverage - Likely to enjoy considerable commercial development
**Cercariae traps**	- Traps could be cheap and easy to deploy in numbers to achieve wide coverage	- Current trap surface areas are small - Laborious if the examination is microscopical, likely high noise backgrounds - Does not allow for specific identification of cercariae found	- Technique requires improved methods for selectively attracting cercariae and to be sure fatty acids and sticky components on traps work as envisioned	- Potential alternative to use of sentinel rodents - May be viewed as an alternative way to collect eDNA so it could be coupled to molecular identification methods
**Robotics**	- A snail-collecting robot is an approach that could revolutionize sample collection and allow for very high coverage	- Costs at present would be prohibitive for routine use in many areas	- Concept is intriguing but much more proof of its practicality and effectiveness required	- Is a concept at this point, might someday play a practical role in surveillance and control efforts
**Oligochromatic dipsticks**	- This is a general approach that could be coupled with several detection methods - It could offer a quick read out at the point of sample collection - Offers standardized format, without need for high tech equipment	- Cost may be prohibitive for widespread use, but this may rapidly trend downward if demand were to increase - Positive readouts may require sequence verification, especially if the signal is equivocal	- Development to accommodate the dipstick format to the sampling of eDNA or snail samples will be required	- It might be particularly suited to locations where the epidemiology is less complex (single snail and schistosome species) such that the meaning of a band would be clearer
**Filtration then molecular characterization**	- See eDNA above as filtration is usually the collection method for this technique - Actual costs of filters and apparatus can be very low with low-tech gravity filter set-ups - Filtrates lend themselves to extraction and further molecular processing	- Samples may be inconsistent in quality because of variation in water turbidity from locations to location - Is labor-intensive and cumbersome to collect water samples and to filter them - Coverage limited by time and labor	- Has already become the first step in eDNA-based molecular protocols - More needed to decide on volumes of water to filter, and development of less labor-intensive collection methods	- See eDNA above

## Discussion

Successful application of any diagnostic technique for the determination of schistosome infection in snails or in the water of natural snail habitats requires accurate knowledge of the species of schistosomes and their snail vectors historically or currently present. A single schistosome and snail species might be involved in some locations, but often multiple *Schistosoma* or potential snail host species are present. For instance, *S*. *rodhaini* might complicate the diagnosis of *S*. *mansoni* in some parts of Africa, but not in South America. *S*. *haematobium* may be especially hard to differentiate from closely related animal schistosomes or their hybrids in some sub-Saharan Africa areas. Multiple species of bulinid snails might be implicated to one extent or another as vectors. Some snail species not susceptible to human schistosome infections like *Planorbella duryi* can easily be mistaken as vector species upon superficial examination [18], potentially resulting in misdirected sampling effort. Familiarity with the literature for a specific area, consultation with parasitologists or malacologists, and examining available keys are all helpful. Submission of voucher specimens of schistosomes and snails to an appropriate museum both for subsequent identification and to provide historical documentation should be encouraged.

Another fundamental reality is that the scale of the task required of the diagnostic technique will vary dramatically. The problems posed for ascertaining if and where schistosomiasis transmission might be occurring on a Caribbean island or in a desert country with few transmission sites are quite different compared to some countries where schistosomiasis transmission potentially occurs across vast geographical areas in almost innumerable freshwater habitats. Clearly, such situations may require different sampling and diagnostic approaches.

Especially in the context of verifying elimination, an all-important consideration is the habitat sampling protocol to be used, a topic requiring much further collective discussion. Given that schistosome transmission is often focal, sampling effort should be guided by available information about past transmission foci and any new evidence (such as positive serology results from recent human surveys) providing relevant locality data. One general goal though, should be to generate many samples from different locations and time points to avoid the possibility of overlooking new or unknown foci of transmission, possibly including ones involving animal reservoirs. Consequently, whatever methods will be chosen to detect positive snails or water samples should be compatible with efficient and low-cost sampling methods that provide realistic coverage to support an accurate assessment of achievement of elimination.

Spear et al. [19] noted, “the development of environmental diagnostics for the infective stage of parasites such as schistosomes is stunningly behind those of other parasites present in water.” In **Table 1**, we identified basic features required of the ideal diagnostic test to answer the stated PICO question. Although the ideal technique does not exist, considerable strides have been made in recent years in devising sensitive and specific detection methods and providing more straightforward methods for extractions, amplifications, and documentation. We are making progress.

As we move forward, we may learn that the application of a combination of diagnostic approaches is inescapable. For example, although frequently maligned, the snail shedding technique has some benefits that may be impossible to replace with even the most sophisticated molecular technique. For one thing, it is *specimen-based*, providing indisputable evidence about the presence of a particular snail vector species and possibly of the schistosome species it is transmitting. The specimens, or portions derived from them, can be used in follow-up molecular analyses and can be vouchered to become part of a more permanent record and facilitate their use in applications we cannot yet anticipate. The shedding method can also provide spatially and temporally explicit information from multiple locations about how common schistosomes might be. It could be argued that familiarity with this method *should* be part of the training of any person working in schistosome environmental diagnostics, simply to reinforce an understanding of the underlying biological realities of the system.

One of the most promising recent developments in schistosome environmental diagnostics is to use as starting material schistosome or snail eDNA as derived from water samples. Suppose eDNA samples are shown not to rapidly decay [20], not to be prone to inhibition and enable sensitive, specific, repeatable analyses across multiple aquatic habitat types.. In that case, it is easy to imagine eDNA as the preferred method of sample collection, perhaps supplemented with occasional snail specimen collection for validation purposes. One great appeal of eDNA samples is the potential for rapidly collecting many samples, each of which effectively integrates a signal over space and time, providing further coverage. Additional improvements to facilitate the collection of eDNA samples are sure to follow, further underscoring this approach’s value.

Once a DNA sample is in hand, then most modern diagnostic approaches proceed to amplify DNA via an ever-growing variety of techniques. This field is moving fast, and what seems best in 2021 may soon be dated. As noted in **Table 3**, there are pros and cons to each. One notable development to go along with the increasing use of eDNA is qPCR-based technology to analyze extracted specimens [20–24]. A quenchable fluorescent probe designed to detect a particular sequence of the target organism of interest is typically included (FRET-qPCR). Significant general advantages for qPCR are that amplification and detection are combined in a single step, it is a mature technique with well-defined protocols and data analysis, it is relatively quantitative in allowing the amount of amplified product to be determined (but requires reference samples and development of a standard curve), costs are relatively low per sample, and it can be set up for high-throughput and multiplexing (perhaps simultaneously detecting schistosome and snail signals in the same sample). It is supported commercially by the active development of new products. Smaller, less expensive qPCR machines are commercially available but are less amenable to high-throughput sampling. Droplet digital PCR has some key advantages over qPCR should eDNA samples prove difficult to analyze: ddPCR is much less subject to inhibition, sensitive to detection of rare targets, and provides absolute quantification.

qPCR and ddPCR offer many advantages, but acquiring and maintaining the instrumentation, procuring reagents in a cost-effective manner, and standardizing protocols among separate labs would remain a challenge. To avoid these problems, and to take advantage of purchasing reagents in bulk and using high-throughput protocols, submission of relatively stable eDNA/DNA samples to one or a few central facilities charged with the responsibility of extracting and amplifying the samples and interpreting the data seems to us to be the way forward. A strong case could also be made for LAMP or other isothermal techniques, including the possibility of having a more widely distributed system of labs employing the technique.

No matter the means of sampling or the technique chosen for detection, some key general points remain. It would be highly desirable from a global elimination program’s standpoint to ensure that the techniques employed have been widely tested on samples collected from many different habitat types on other continents. Standardization with respect to extraction methods, particular reagents or kits used, means to test for or prevent inhibition of detection reactions, amplification protocols, and data interpretation and presentation are all highly desirable.

In the case of amplification-based techniques such as PCR/qPCR strict precautions must be taken to prevent contamination of the lab environment with amplicons of the target species to avoid the recording of false positives. The inclusion of both positive and negative controls and standards in all protocols is essential. Aliquoting of key reagents is vital to minimize contamination risk. Physical separation of separate steps in preparation of amplification reactions, if possible, is also essential. If qPCR, ddPCR, or other complex equipment prone to noise-to-signal inaccuracies are to be involved, regular and careful calibration of the machines is critical.

With respect to testing the primers used for various techniques, to avoid misleading results, there is a need to be sure 1) the *closest* and *most common* relatives of the target species are tested, and 2) that spurious cross-reactions even with parasite species that are not close relatives of the target species are excluded as a possibility. Testing against *local* co-occurring snail and trematode species is preferred as these are the species likely to cause cross-reactions and false positives to occur [22]. Having additional genome sequences for the trematode species most likely to co-occur with targeted schistosome species, either in the same snail species or the same environment, would be helpful. It would help eliminate the possibility of designing primers based on schistosome genes or genomic repeats that quite unexpectedly share sequence similarity with other trematodes. Extraneous trematodes may be more of a diagnostic complication for detecting schistosomes in snails than presently envisioned because of the tendency of groups like echinostomes, xiphidiocercariae, and strigeids to encyst as metacercariae within field snails. Similar considerations apply to eDNA samples taken from environments supporting diverse trematode faunas, which is often the case.

Primer design often relies on *in silico* predictions to limit cross-reactivity, which may have pitfalls [25], underscoring the need for follow-up confirmation. Sanger or high-throughput amplicon sequencing with comparison to sequences in GenBank [26,27] will be particularly crucial during elimination and surveillance operations to ensure positive results that are recorded by a band, Ct value, or a color change in a tube definitively belong to the schistosome or snail species responsible for the positive sample. Cross-reactions could lead to erroneous conclusions about the persistence of transmission or lead to misidentification of hot spots, in turn leading to erroneous targeting of special control efforts and wasting limited resources.

It is also important to rule out the effect of inhibitors in diminishing or precluding amplification. Inhibitors can originate from the sample or can be introduced during extraction. The standard method to deal with inhibitors is through control reactions spiked with known amounts of a reliably amplifiable non-target sequence. By comparing the quantity of non-target detected to the originally spiked amount, the level of inhibition can be determined in any given sample. This helps to guard against obtaining false negatives with unknown samples. If the presence of inhibitors is deemed a persistent problem, then sample treatments may be required, or the use of ddPCR may then be preferred.

Regarding the molecular targets that have been used for schistosome detection (see [28] for one list), they are quite understandably typically based on sequences expected to be represented many times in a single schistosome cell, thereby increasing the ability to sensitively detect a signal. They include mitochondrial markers (such as 16S rRNA, *Cox1*, *Cox3*, *Nad5* (*ND5*) [29]), or nuclear sequences including portions of the rRNA complex (18SrRNA, intergenic 28S-18S spacers) and repetitive sequences such as Sm1-7 and DraI [12]. Many of these sequences have also been used to detect schistosomes in human samples such as plasma [30], however snails and environmental samples pose added challenges due to presence of a multitude of potential competing sequences, that as noted above might have surprising homologies with schistosome target sequences. Certainly, there is additional scope for development of better target sequences that might avoid cross-reaction or resist degradation, but it is also important to more thoroughly test the primers we have. One recent comparative study found primer sets targeting *ND5* or 28S to work better than primers targeting a repeat element [31], the latter potentially also favoring amplification of unknown products from other complex genomes also present in extracted samples.

Particularly needed for eDNA-water based approaches are studies comparing different primer sets under a diversity of realistic field conditions in which known quantities of schistosome specimens or DNA are present, preferably undertaken by the same research team. Such controlled comparative studies should include considerations of how eDNA moves and degrades over time. Included in the comparisons would be considerations of different extraction techniques and downstream amplification protocols such as LAMP and qPCR. An important consideration is tailoring primers to the amplification method: gel-based PCR detection can accommodate long amplicons which can be made highly specific, however qPCR and ddPCR have better performance using shorter amplicons in the 70–200 bp range, limiting the potential regions to amplify. Considering the plethora of genomic resources [32] and molecular targets already found for schistosomes, it is likely that the limiting factor to the identification of the best targets to use is the lack of comprehensive comparative studies rather than the lack of useful molecular targets.

It will be tempting to pool samples, especially when in a surveillance program, it becomes clear that most samples collected and analyzed prove to be negative. Pooling could allow expanded coverage and sharply lower expenses. For some snails like the tiny *Oncomelania* involved in *S*. *japonicum* transmission, this may prove more feasible, as some of the papers examined suggest (one cercaria can be detected per 1000 uninfected snails). For larger snails like *Bulinus* or *Biomphalaria*, pooling may prove more complicated, especially if rare and/or prepatent infections are represented among the pooled snails. Recognizing that a trade-off is involved in the pooling of samples, one way to check the ability of pooling to detect small schistosome signals is to spike some of these samples with various doses of parasites to see if they reliably detect positives. Similar considerations apply to pooled water samples.

For studies based on detection of eDNA from habitats, it is important to recognize that positive signals from filters could be derived from cercariae that were intact and alive at the time of sampling or from environmental DNA no longer associated with living cercariae or any other life cycle stage. Positive signals from both sources are valuable but tell different stories. If positive signals from living cercariae are being detected, then the techniques convey valuable information that infections are ongoing and where they are being acquired. If the signals originate from eDNA, they are more indicative of a signal integrated over time. The length of the persistence of eDNA signals is bound to be variable, from days to weeks [33], which will influence the extent to which signals may be distributed within a habitat. Developing techniques to detect eRNA, indicative of the presence of living specimens, may prove to be useful in some contexts.

## Conclusions

Some important knowledge gaps going forward are:

1. We are not necessarily there yet with respect to identifying the best schistosome sequences to target for amplification. The optimum length of the target to be sequenced may well depend on the method of amplification. Further comparative studies to assess existing primers [31] and more testing to improve specificity with respect to the realistic identification challenges faced in a particular location are needed.
2. To field-test the most promising sample collection, extraction methods, primers, and detection methods in multiple locations, possibly with standardized spiked samples. This would be undertaken preferably in a coordinated, comparative fashion, under centralized supervision such that quality control and standardization can be promoted. This would help identify ways to reduce costs and identify the most suitable combination of techniques for broader application.
3. Further emphasis on the development of eDNA based methodology is warranted to determine: temporal stability of amplifiable signals in the water column; how far eDNA might be dispersed and still be detected; if robust and consistent signals be retrieved from complex tropical aquatic ecosystems, and; if the samples be easily shipped elsewhere for analysis?
4. Ideally, our diagnostic tool kit would eventually include approaches that might allow differentiation among live or dead specimens and different life cycle stages of the same species, for instance, differentiating between miracidia (indicative of contamination) or cercariae (indicative of the potential for infection) and determining if infections in snails derive from humans or reservoir hosts.
5. We must remain open-minded for dramatic new approaches that rely on robotics for sample collection/processing or for self-contained microfluidics devices that, if proven effective and made inexpensive, could solve many of the coverage and standardization problems.
6. Inducements to encourage more researchers to participate and communicate with one another to develop standardized new diagnostic techniques should be provided.

## Supporting information

S1 Table Criteria used for the evaluation of each reference, and the extraction summaries for each reference evaluated–presented in Excel format. (XLSX)

S2 Table Specificity here is defined as the ability of the method to correctly identify specimens without signal/infection (true negatives); Sensitivity, the true positive rate (specimens with infection/signal); Sensitivity to input: In most of the studies we looked at this was the proxy used for sensitivity as the minimum amount of sample required which in turn determines the ability of the method to detect true positives. This is reported as either DNA amount or number of cercariae. n *=* to the number of studies used to obtain the numbers reported.
(DOCX)
