# Supplementary material for: Detecting and identifying Schistosoma infections in snails and aquatic habitats: A systematic review
Source: PLoS Negl Trop Dis. 2021 Mar 24;15(3):e0009175. doi: 10.1371/journal.pntd.0009175 (PMC8021170; doi:10.1371/journal.pntd.0009175)
Supplement: S2 Table — This is reported as either DNA amount or number of cercariae. n = to the number of studies used to obtain the numbers reported. (DOCX) [file pntd.0009175.s002.docx]

| **Method** | **Specificity** | **Sensitivity** | **Sensitivity to input** |
| --- | --- | --- | --- |
| Direct Shedding | Insufficient Data | 25%-100%; n=3 | Insufficient Data |
| Snail crushing | Insufficient Data | Insufficient Data | Insufficient Data |
| ELISA/immunodetection | 100%; n=2 | 88%-100%; n=4 | Insufficient Data |
| Biochemical analysis | Insufficient Data | Insufficient Data | Insufficient Data |
| DNA hybridization /DOT BLOT | Insufficient Data | 100%; n =1 | 1-5 ng DNA; n=2 |
| **PCR** |  |  |  |
| Conventional PCR | 90%-100%; n=3 | 100%; n=1 | 1fg - 10 ng DNA; n=17 |
| PCR with Restriction digestion | Insufficient Data | Insufficient Data | Insufficient Data |
| RAPD PCR | Insufficient Data | Insufficient Data | Insufficient Data |
| Repeat Sequence PCR | Insufficient Data | Insufficient Data | 10 fg - 1 pg DNA; n=4 |
| Nested PCR | Insufficient Data | 80%-92%; n= 2 | 0.1 fg - 1 fg DNA; n =2 |
| Multiplex PCR | 70%; n =1 | Insufficient Data | 1 pg - 0.78 ng DNA; n=2 |
| qPCR | 80%-85.7%; n=2 | 93%; n=1 | 2 fg - 14 fg DNA; n =3 |
| FRET-PCR | 100%; n =1 | Insufficient Data | 0.004 ng DNA; n =1 |
| ddPCR | 100%; n =1 | Insufficient Data | 0.05 fg DNA; n =1 |
| **Isothermal Amplification Techniques** |  |  |  |
| LAMP | 86.67% - 94%; n=2 | 96.7% -100%; n=2 | 70.1 fg - 0.01 ng DNA; n=7 |
| Microfluidics LAMP | Insufficient Data | Insufficient Data | 0.5 fg DNA; n =1 |
| Recombinase polymerase amplification | Insufficient Data | Insufficient Data | 100 fg DNA; n=1 |
| **Water-based detection** |  |  |  |
| Filtering then direct exam of filter | Insufficient Data | 30% - 93.75 %; n=4 | 4 cercaria / 100 L; n =1 |
| Sentinel rodents | Insufficient Data | Insufficient Data | Insufficient Data |
| Sentinel snails | Insufficient Data | Insufficient Data | Insufficient Data |
| eDNA | 53% - 95%; n = 3 | 75%-95%; n =3 | 1 cercaria / 16-20 L |
| Cercariae traps | Insufficient Data | Insufficient Data | Insufficient Data |
| Robotics | Insufficient Data | Insufficient Data | Insufficient Data |
| **Others*** |  |  |  |
| Oligochromatic dipstick | Insufficient Data | Insufficient Data | 10 fg DNA; n =1 |
| Filtration then molecular characterization | Insufficient Data | 50%; n =1 | 1 cercaria / 10-16; n =2 |
|  |  |  |  |
